# The combination of microfracture with induction of Wnt / β- Catenin pathway, leads to enhanced cartilage regeneration

**DOI:** 10.1186/s13018-019-1484-3

**Published:** 2019-12-11

**Authors:** Nikitas P. Schizas, Olga Savvidou, Kalliopi Diamantopoulou, Stamatios Papadakis, Panayiotis J. Papagelopoulos, Ioannis K. Triantafyllopoulos

**Affiliations:** 1 Laboratory for the Research of Musculoskeletal System, Medical School, National and Kapodistrian University of Athens, 10 Athinas Street, 14561 Kifissia, Greece; 2 First Department of Orthopaedics, Athens University Medical School, National and Kapodistrian University of Athens, School of Medicine, 41 Ventouri Street, 15562 Holargos, Athens Greece; 3 Pathology Department, KAT Hospital Kifissia, 2 Nikis Street, 14561 Kifissia, Athens Greece; 4 2nd Department of Orthopaedic Surgery, KAT Hospital Kifissia, 2 Nikis Street, 14561 Kifissia, Athens Greece

**Keywords:** Microfracture, Cartilage repair, Fibrocartilage, Wnt/β-catenin pathway, Lithium carbonate

## Abstract

**Introduction:**

Microfracture does not lead to complete healing of full-thickness cartilage defects. The aim of this study was to evaluate the effect of modifying Wnt/β-catenin signaling following microfracture, on the restoration of a full-thickness cartilage defect in a rabbit model. The modification of the canonical Wnt pathway was achieved through per os administration of lithium carbonate, which is an intracellular inhibitor of glycogen synthase kinase 3-β (Gsk3-β) and therefore induces Wnt/β-catenin signaling.

**Materials and methods:**

Full-thickness cartilage defects of 4 mm in diameter were created in the patellar groove of the right femurs of 18 male New Zealand white rabbits. The rabbits were divided into three groups of six (*n* = 6) based on post-surgery treatment differences, as follows: microfracture only (group 1), microfracture plus lithium carbonate 7 mM in the drinking water for 1 week (group 2), microfracture plus lithium carbonate 7 mM in the drinking water for 4 weeks (group 3). All animals were sacrificed 9 weeks after surgery. The outcome was assessed histologically, by using the International Cartilage Repair Society (ICRS) visual histological scale. Immunohistochemistry for type II collagen was also conducted.

**Results:**

Statistical analysis of the histological ICRS scores showed that group 3 was significantly superior to group 1 in four out of six ICRS categories, while group 2 was superior to 1 in only two out of six.

**Conclusion:**

The combination of microfracture and systematic administration of lithium carbonate 7 mM for 4 weeks shows statistically significant superiority in four out of six ICRS categories compared with microfracture only for the treatment of full-thickness cartilage defects in a rabbit experimental model.

## Introduction

Articular cartilage has a low intrinsic reparative capacity [1]. Marrow-stimulating procedures are indicated for treating small, up to 4-cm^2^ full-thickness cartilage defects. In these procedures, perforation to the subchondral bone allows blood and marrow-derived cells to fill the defect and a blood clot is formed. The subsequent wound repair cascade finally leads to the formation of vascularized granulation tissue and the proliferation of pluripotent mesenchymal progenitor cells with a capacity to differentiate into multiple mesenchymal cell types [2]. In the first days following subchondral perforations, fibrinous arcades are formed across the surface of the defect. The scaffold they create serves to guide mesenchymal cell ingrowth along the long axes. Afterward, undifferentiated mesenchymal cells progressively differentiate in fibroblasts, osteoblasts, articular chondroblasts, and chondrocytes. Finally, new bone forms into the deeper zones and fibrocartilage into the superficial zones of the newly formed tissue [3–5].

Cartilage formation is the desirable outcome in osteochondral lesions, but it is quite unwelcome as a final result of the fracture healing process. An important observation made in fracture nonunion is the presence of cartilage between the bone ends, associated with the formation of fibrous tissue and minimal bone regeneration [6–8]. During endochondral ossification, cartilage formation is an important intermediate step of osteogenesis. In several models of fracture nonunion, cartilage formation is not followed by efficient endochondral ossification, while fibrous tissue forms instead. The transition from cartilage to bone is a process which is regulated by locally produced growth factors [9, 10]. In a study by Kwong et al. (2009), it was shown that imbalance in the expression of bone morphogenetic proteins (BMPs) and BMP inhibitors within cartilaginous areas of developing non-unions may account for their reduced bone formation ability [11].

These findings imply that, if microfracture regenerative procedure was considered as a special case of fracture repair, several pathways could be targeted in an attempt to promote the recruitment of progenitor cells towards the chondroblast instead of osteoblast lineage during endochondral ossification. Both fracture healing and endochondral bone formation are directly regulated by BMPs [12, 13], fibroblast growth factor 2 (FGF-2) [14], Wnt proteins and Wnt signaling antagonists [15, 16]. Several of these morphogenetic processes participate in interactive feedback loops, including the interplay between BMPs and Wnt signaling proteins [17, 18]. Specifically in the case of the canonical Wnt pathway, β-catenin signaling has different effects at different stages of bone repair. Early in the process, it controls the ratio of osteoblasts and chondrocytes made from the pluripotent mesenchymal cells. Later on, β-catenin promotes the differentiation of osteoblasts [19].

Key regulator of the canonical Wnt pathway is glycogen synthase kinase 3-β (Gsk3-β). In the absence of appropriate Wnt ligands, a destruction complex comprising Axin and adenomatous polyposis coli (APC) mediates the phosphorylation of β-catenin by Gsk3-β, which leads cytosolic β-catenin to degradation by the proteasome. The presence of certain agents such as lithium (Li) has been shown to induce the phosphorylation of Gsk3-β, rendering the kinase inactive. This is followed by the reduction of Gsk3-β activity and accumulation of cytoplasmic β-catenin, which will function as a co-factor of TCF/LEF transcription factors to induce expression of Wnt target genes. Gsk3-β activity is required for both chondrocyte and osteoblast differentiation [20]. Activation of Wnt signaling by means of Gsk3-β antagonist lithium in cell lines and mesenchymal stem cells has the ability to stimulate the expression of chondrogenic markers [21].

Τhe aim of this study was to evaluate the effect of modifying Wnt/β-catenin signaling following microfracture, on the restoration of a full-thickness cartilage defect in a rabbit model. The modification was achieved through per os administration of lithium carbonate, which is an intracellular inhibitor of Gsk3-β and therefore induces Wnt/β-catenin signaling. Finally, Gsk3-β inhibition causes β-catenin accumulation inside the nucleus of cells, where the transcription of target genes is triggered [19].

## Materials and methods

Cartilage defects were experimentally created in the right trochlear groove of 18 male New Zealand white rabbits, weighing between 3.0 and 3.6 kg and 16 weeks of age. All animal procedures were approved by the Veterinary Policy Committee of the prefecture of Attica Greece and the Scientific Council of KAT General Hospital Kifissia Greece. All procedures were conducted in the experimental surgery of the Laboratory for the Research of Musculoskeletal Disorders, Medical School, National and Kapodistrian University of Athens, Greece.

### Surgical procedure

**Fig. 1:**
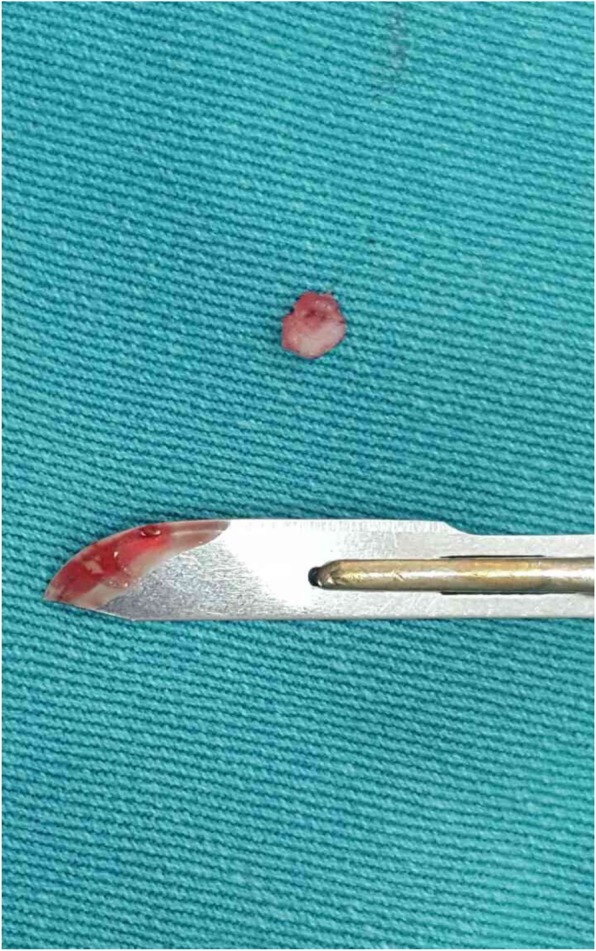
Full-thickness cartilage defect, 4 mm in diameter

**Fig. 2:**
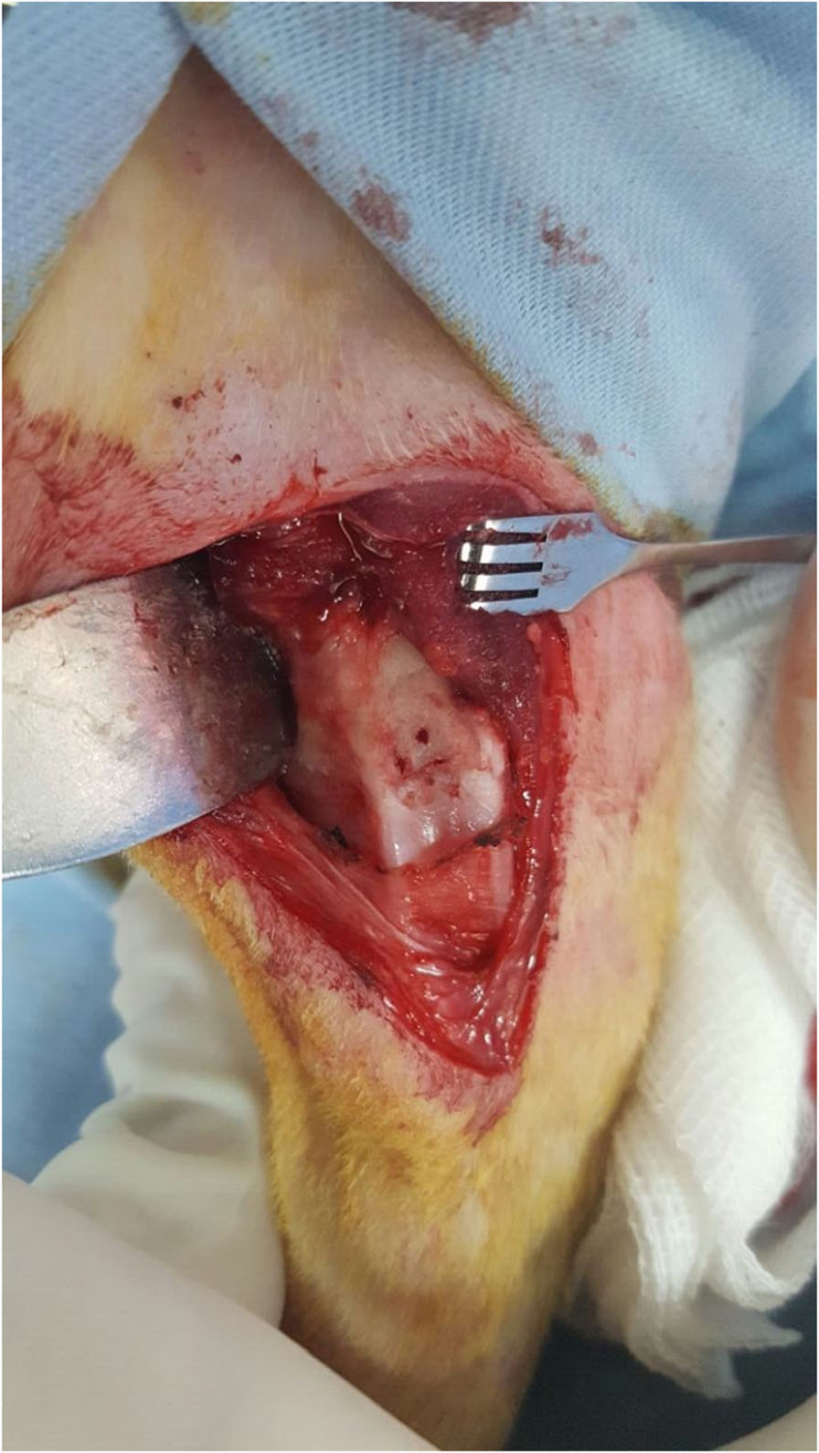
Four microfracture perforations were created, in a distance of 1.0–1.5 mm from each other, within a12.56 mm^2^ cartilage defect surface

Each rabbit was anesthetized with an intramuscular injection of Midazolam 2 mg/kg (Dormicum) and Ketamine 30 mg/kg (Imalgene). In addition, anesthesia was maintained by slowly administering 30 mg/kg Thiopental through an auricular vein. The rabbit was placed in the supine position, and a medial parapatellar approach was used to operate on the right knee joint. Then, the patella was dislocated laterally to expose the articular surface. A full-thickness chondral defect (4 mm in diameter) was generated at the center of the trochlear groove by using an osteochondral transplantation system. The defect was completely debrided of its calcified cartilage layer. Then microfracture was performed with a 1.0-mm Kirschner wire with a stopper at 2 mm from the tip, in order to control the penetration depth. Within the chondral defect, 4 microfracture perforations were created, at a distance of 1.0–1.5 mm from each other (Figs. 1 and 2).

### Postoperative treatment

After surgery, rabbits were allowed to assume free ambulation in their cages. Their operated limbs were allowed to fully weight-bear, and their general health status was regularly monitored by a veterinarian.

### Lithium carbonate administration

The rabbits were randomly divided into the following three groups: microfracture only (group 1), microfracture plus lithium carbonate of 7 mM in the drinking water for 1 week (group 2), microfracture plus lithium carbonate of 7 mM in the drinking water for 4 weeks (group 3). Lithium carbonate was administered orally by adjusting the drinking water to a concentration of 7 mM. We obtained blood specimens from an auricular vein at weeks 1 and 4 and measured lithium concentrations. This treatment protocol resulted in serum lithium concentrations of 0.40 ± 0.20 mM/L (mean ± SEM) which were relatively stable at weeks 1 and 4. These serum levels are slightly lower than the range of 0.6–1.2 mM/L, concentrations achieved and maintained during lithium therapy in human subjects [22]. All rabbits were monitored daily for clinical symptoms. They were sacrificed 9 weeks after surgery, and the right femur was harvested. The outcome was assessed histologically, by using the International Cartilage Repair Society (ICRS) visual histological scale. Immunohistochemistry for type II collagen was also conducted.

### Histologic evaluation

The specimens were fixed in 10% neutral w/v phosphate buffer 0.05 M and decalcified in 2% nitric acid and afterward 4% formic acid. Afterward, they were dehydrated with serial ethanol, embedded in paraffin, and cut into 3-μm sections. (FINESSE ME+, Thermo Scientific). The sections were stained with toluidine blue, hematoxylin–eosin and Weigert–Van Gieson stains and viewed under a light microscope. They were blindly scored by two different investigators according to the International Cartilage Repair Society (ICRS) visual histological scale. Immunohistochemistry for collagen type II was performed in all groups, using the EnVision Flex kit and the Autostainer Link (DAKO).

### Statistical analysis

Data were expressed as median (IQR) for quantitative variables and as percentages for qualitative variables.

The comparison of variables between 3 groups was performed using the Kruskal–Wallis test. Pairwise comparisons were performed using the Dunn adjusted by the Benjamini–Hochberg FDR test.

All tests were two-sided, and statistical significance was set at *p* < 0.05. All analyses were performed using the statistical package SPSS vr 21.00 (IBM Corporation, Somers, NY, USA).

## Results

**Fig. 3:**
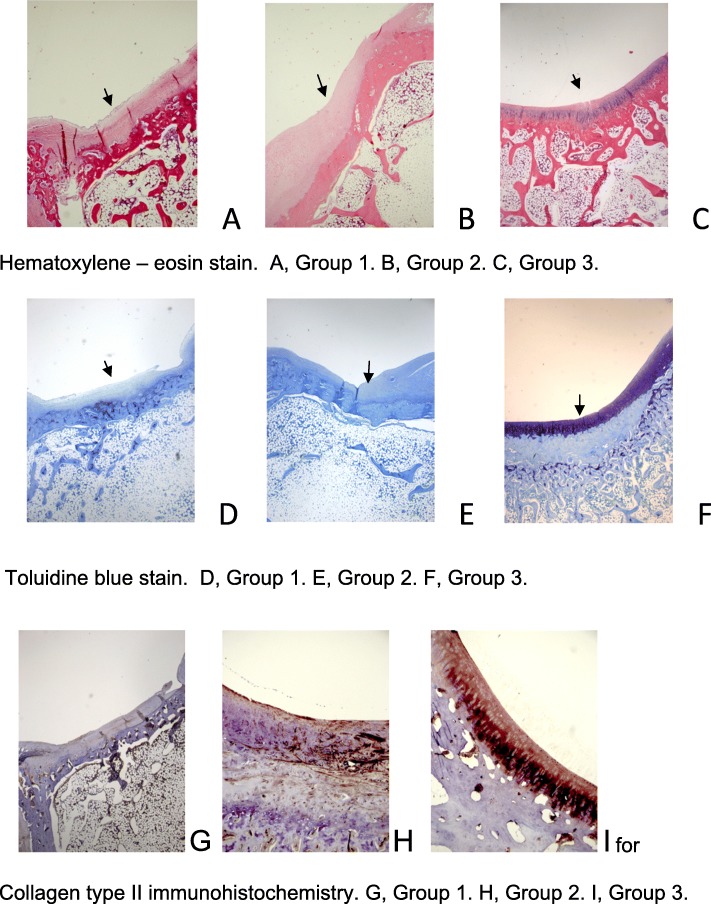
**a**–**i** Histology for group 1 revealed the irregular surface of the newly formed tissue, disorganized cell distribution, and the presence of a fibrocartilage matrix (**a**, **d**). Group 2 showed a more regular surface; the matrix was composed of both hyaline cartilage and fibrocartilage, while cell distribution would range from disorganized to mixed columnar and cluster (**b**, **e**). Group 3 demonstrated a regular surface, with hyaline matrix mixed with fibrocartilage in some areas, but with chondrocytes distributed in a more organized pattern (**c**, **f**). Arrows highlight the surface. Immunohistochemistry in the control group was consistent with weakly detectable collagen type II fibers. In group 2, they were detectable but would follow an irregular pattern, while group 3 revealed orderly oriented collagen type II fibers (**g**–**i**)

The histological sections of group 1were characterized by an irregular surface of the newly formed tissue, the cell distribution was disorganized and they presented a fibrocartilage matrix (Fig. 3a, d). Group 2 showed a more regular surface, the matrix was composed of both hyaline cartilage and fibrocartilage, while cell distribution would range from disorganized to mixed columnar and cluster (Fig. 3b, e). The histological findings for group 3 demonstrated a regular newly formed tissue surface, with a hyaline matrix mixed with fibrocartilage in some areas, but with chondrocytes distributed in a more organized pattern compared to the other groups (Fig. 3c, f). Cartilage mineralization was normal in all groups. The International Cartilage Repair Society (ICRS) visual histological scale was estimated for each one of the laboratory animals. Animals 3 and 11 were excluded from the study due to histological findings of osteomyelitis, which notably did not result in clinical manifestation. Immunohistochemistry for collagen type II in group 3 revealed orderly oriented fibers, while in group 2 they would follow an irregular pattern. In the control group, collagen type II fibers were weakly detectable (Fig. 3g–i).

**Fig. 4:**
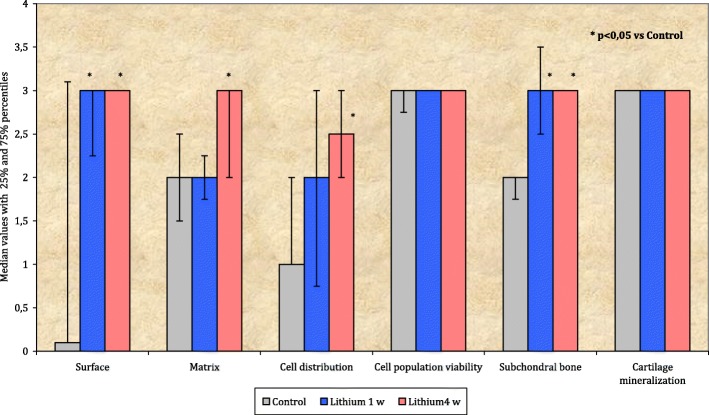
Comparison of histological parameters between groups

Group 3 (lithium intake for 4 weeks) presented statistically significant higher values of surface (*p* = 0.028), matrix (*p* = 0.022), cell distribution (*p* = 0.029), and subchondral bone (*p* = 0.004) compared with the control group. Group 2 (lithium intake for 1 week) presented statistically significant higher values of surface (*p* = 0.028) and subchondral bone (*p* = 0.050) compared with the control group (Fig. 4).

## Discussion

Microfracture initiates a healing process that fills cartilage defects with fibrocartilage repair tissue. Studies aiming at adjuvant treatments to microfracture technique have three targets: (a) to optimize the environment of the clot created for the proliferation of the marrow-derived mesenchymal stem cells (MSCs), this being achieved through the use of membranes or scaffolds that cover the clot and therefore protect the cells contained within [23, 24], (b) to utilize the effect of pharmaceutical agents that have chondroprotective properties, or can induce chondrogenesis [25–28], (c) to biologically enhance the microfracture clot, by adding growth factors into it, in order to improve the quality and quantity of the regenerative tissue [27, 29–33]. Furthermore, the combination of more than one adjuvant treatments simultaneously could offer an even better final outcome [34–37].

One of the morphogenetic pathways regulating fracture and consequently microfracture healing is the Wnt/β-catenin. The interplay between several components of the pathway causes β-catenin accumulation inside the nucleus of cells, where the transcription of target genes is triggered. The Wnt/β-catenin pathway is also involved in both pre-natal and adulthood skeletal development, so several studies have investigated the potential of Gsk3-β inhibition upon chondrogenic differentiation [19]. The findings, however, remain inconclusive.

In an animal experimental model, intra-articular injection of GIN, a Gsk3-β inhibitor in rat knee joints induced nuclear accumulation of β-catenin in chondrocytes and was associated with surface fibrillation, a decrease in glycosaminoglycan expression and chondrocyte hypocellularity. These results suggest that, by downregulating β-catenin, Gsk3-β preserves the chondrocytic phenotype and is involved in the maintenance of the cartilage extracellular matrix. Short term β-catenin upregulation in cartilage secondary to Gsk3-β inhibition may be sufficient to induce osteoarthritis-like features in vivo [38].

On the contrary, Gsk3-β inhibitors appeared to significantly enhance cartilage matrix production in chondrogenic cultures. In this in vitro study, MSCs were derived from human marrow aspirates, centrifuged into small aggregates, and provided with a chondrogenic medium supplemented with either lithium or SB216763, which is another Gsk3-β inhibitor. The addition of either SB216763 or lithium to cultures significantly enhanced matrix glycosaminoglycan concentrations [39].

Additionally, in an in vitro pellet culture model, a binary SiO2–Li2O solution gel–derived glass was utilized for the controlled delivery of lithium. Dissolution products that contained 5 mM lithium and 3.5 mM silicon induced chondrogenic differentiation and hyaline cartilaginous matrix formation without the presence of growth factors such as TGF-β [40].

The main findings of our study are the following: (1) Induction of the Wnt/β-catenin signaling pathway for 4 weeks after application of microfracture results in significantly statistical superiority in four out of six histological ICRS categories. (2) Immunohistochemistry in group 3 is consistent with orderly oriented collagen type II fibers, while in group 2 they would follow an amorphous entangled pattern. (3) Induction of the pathway during only the first week post microfracture was not sufficient to exert the beneficial effect of the 4-week treatment upon the histological properties of the repair tissue. On the other hand, it demonstrated statistically significant higher ICRS values of surface and subchondral bone compared with the control group.

In the present study, the chondrogenic differentiation effect of lithium as a Gsk3-β inhibitor revealed by culture studies was exerted on the microfracture clot. The wound repair cascade initiated in the clot and the proliferation of pluripotent mesenchymal progenitor cells with a capacity to differentiate into multiple mesenchymal cell types offer a substrate with healing potential. In the case of intra-articular injection of GIN in rat knees, given the low intrinsic cartilage reparative capacity, there is a lack of substrate for the chondrogenic differentiation effect of lithium. The aforementioned combination of healing potential through microfracture and chondrogenic differentiation through lithium may justify the results of our study.

The tissue produced in articular lesions after microfracture presents good short term clinical results. However, fibrocartilage is mechanically weaker than hyaline cartilage and degenerates easily. Deterioration tends to occur beyond 2 years as the fibrocartilage breaks down [41]. A limitation of the present study is that the follow-up time could not demonstrate whether the repair is long-lasting. Future work incorporating a 6-month or 1-year follow up time point is needed in order to determine the long-term outcomes of this adjuvant therapy.

Regarding other limitations of the study, it is clear that lithium administration requires monitoring of the blood levels, as is routinely practiced for patients who receive it as bipolar disorder treatment. Ensuring lithium concentration that does not exceed therapeutical levels is of key importance, as it minimizes side effects. Notably, in our study, no adverse events due to lithium treatment were noted. Additionally, lithium is widely known as a mental disorder medicine, a fact that might compromise patient compliance, where they intended to receive it for another purpose. Such problems could be overcome by the utilization of an anti-sclerostin antibody as Wnt/β-catenin signaling enhancer.

On the other hand, lithium is an inexpensive and rather safe medication well studied for many years. Additionally, systemic administration ensures that, through blood flow, lithium is transported inside the forming clot itself and therefore exerts its action effectively, while a reagent that would require intra-articular injection would form an interface with the surface of the clot only.

Considerations regarding the design of our experimental model include the dimensions of the full-thickness cartilage defect, as well as the tools used to generate microfractures. In order to simulate clinical practice, we created four holes of 1 mm diameter in a 12.56-mm^2^ cartilage defect, which is 25% of the area. According to the literature, the percentage found in clinical practice is 20–40% [42]. Additionally, given that our experimentally generated defect is perhaps 10 times smaller than a typical 1.5-cm^2^ human lesion the scaling factor of 3 for linear dimensions was applied to choose the proper tool. Finally, the tool used was 1 mm in diameter while the clinical tool is 3 mm.

## Conclusion

Based on the findings of the present study, the induction of the Wnt/β-catenin signaling pathway may augment the microfracture derived repair tissue when treating full-thickness cartilage defects. Certain limitations must be dealt with in order to utilize this combined, one stage treatment at the clinical level. More experimental studies are required in order to determine the optimal interaction between the microfracture regenerative procedure and the intrinsic induction of the Wnt/β-catenin pathway. Other canonical Wnt signaling participating molecules may be targeted, but finally, the conclusion of the present experimental study is that inducing the pathway after microfracture may be an effective adjuvant therapy for treating full-thickness cartilage defects.

## Data Availability

All data generated or analyzed during this study are included in this published article (and its supplementary information files).

## Abbreviations

APC: Adenomatous polyposis coli
BMPs: Bone morphogenetic proteins
FGF-2: Fibroblast growth factor 2
GIN: 3-[9-Fluoro-2-(piperidine-1-carbonyl)-1,2,3,4-tetrahydro-[1, 4] diazepino [6,7,1-hi]indol-7-yl]-4-imidazo [1,2-a]pyridin-3-yl-pyrrole- 2,5-dione
Gsk3-β: Glycogen synthase kinase 3-β
ICRS: International Cartilage Repair Society
LEF: Lymphoid enhancer–binding factor
Li: Lithium
MSCs: Mesenchymal stem cells
TCF: T cell factor
